# Preoperative staging of perforated diverticulitis by computed tomography scanning

**DOI:** 10.1007/s10151-012-0853-2

**Published:** 2012-06-30

**Authors:** M. P. M. Gielens, I. M. Mulder, E. van der Harst, M. P. Gosselink, K. J. Kraal, H. T. Teng, J. F. Lange, J. Vermeulen

**Affiliations:** 1 Department of Surgery, Maasstad Hospital, P.O. Box 9100, 3007 AC Rotterdam, The Netherlands; 2 Department of Surgery, Erasmus University Medical Centre, P.O. Box 2040, 3000 CA Rotterdam, The Netherlands; 3 Department of Radiology, Erasmus University Medical Centre, P.O. Box 2040, 3000 CA Rotterdam, The Netherlands; 4 Department of Radiology, Maasstad Hospital, P.O. Box 9100, 3007 AC Rotterdam, The Netherlands; 6 Department of Surgery, Admiraal De Ruyter Hospital, P.O. Box 3200, 4380 DD Vlissingen, The Netherlands

**Keywords:** Perforated diverticulitis, Computed tomography scanning, Hinchey classification

## Abstract

**Background:**

Treatment of perforated diverticulitis depends on disease severity classified according to Hinchey’s preoperative classification. This study assessed the accuracy of preoperative staging of perforated diverticulitis by computerized tomography (CT) scanning.

**Methods:**

All patients who presented with perforated diverticulitis between 1999 and 2009 in two teaching hospitals of Rotterdam, the Netherlands, and in addition had a preoperative CT scan within 24 h before emergency surgery were included. Two radiologists reviewed all CT scans and were asked to classify the severity of the disease according to the Hinchey classification. The CT classification was compared to Hinchey’s classification at surgery.

**Results:**

Seventy-five patients were included, 48 of whom (64 %) were classified Hinchey 3 or 4 perforated diverticulitis during surgery. The positive predictive value of preoperative CT scanning for different stages of perforated diverticulitis ranged from 45 to 89 %, and accuracy was between 71 and 92 %. The combination of a large amount of free intra-abdominal air and fluid was strongly associated with Hinchey 3 or 4 and therefore represented a reliable indicator for required surgical treatment.

**Conclusions:**

The accuracy of predicting Hinchey’s classification by preoperative CT scanning is not very high. Nonetheless, free intra-abdominal air in combination with diffuse fluid is a reliable indication for surgery as it is strongly associated with perforated diverticulitis with generalized peritonitis. In 42 % of cases, Hinchey 3 perforated diverticulitis is falsely classified as Hinchey 1 or 2 by CT scanning.

## Introduction

Diverticular disease has become more prevalent in Western countries [1]. About 10–25 % of individuals with diverticulosis will develop symptomatic diverticulitis, and of these, 15 % will develop significant complications, such as perforation [2]. Although the absolute prevalence of perforated diverticulitis complicated by generalized peritonitis is low, its importance lies in the significant postoperative mortality, ranging from 4 to 26 % regardless of the surgical strategy selected [3, 4]. The optimal treatment for perforated diverticulitis is still a matter of debate [5].

Optimal treatment strategies are based on disease severity as classified by Hinchey (Table 1) [6]. Today, a conservative treatment with antibiotics (and abscess drainage) is advocated for Hinchey 1 and 2 perforated diverticulitis [7]. Patients presenting with perforated diverticulitis with generalized peritonitis (Hinchey 3 and 4) should undergo emergency surgical treatment. Laparoscopic peritoneal lavage without resection of the affected bowel segment in patients with purulent peritonitis (Hinchey 3) appears to diminish the morbidity and improve outcome [8–10], whereas acute resection should be performed in patients with gross fecal peritonitis (Hinchey stage 4) [9].

**Table 1:** The modified Hinchey classification of perforated diverticulitis

Hinchey classification	Clinical features
0	Mild clinical diverticulitis
1	
a	Confined pericolic inflammation or phlegmon
b	Confined pericolic abscess
2	Pelvic, intra-abdominal, or retrocolic abscess
3	Generalized purulent peritonitis
4	Generalized fecal peritonitis

In the original Hinchey classification, Hinchey 1a and 1b were combined

Unfortunately, (the modified) Hinchey’s classification is based on clinical findings during surgery. Ideally, one should be informed about the severity of the disease to optimize treatment strategy. Today, computed tomography (CT) scanning is the modality of choice in the assessment and management of diverticulitis with its high sensitivity and specificity [11–15]. With CT-guided percutaneous abscess drainage (PCD), it has also become an important therapeutic modality [11–16]. The CT-based classification by Hansen–Stock can be used as a classification system and accounts for asymptomatic diverticulosis as well as complicated diverticulitis in different stages, including perforation [17]. Nevertheless, the degree of peritonitis—and hence the severity of disease—in perforated diverticulitis can be represented best by the modified Hinchey’s classification.

The aim of this study was to assess the accuracy of preoperative CT scanning in predicting the stage of severity of perforated diverticulitis. The CT findings are compared with the clinical findings during surgery classified according to the Hinchey classification [6].

## Materials and methods

All patients who underwent emergency surgery for perforated diverticulitis between January 1999 and January 2009 at the Erasmus University Medical Centre and Maasstad Hospital in Rotterdam, the Netherlands, were selected from computerized surgery registration databases. After patient selection was completed, predetermined parameters were extracted from medical records and the computerized patient’s registration databases. The indication for surgery was based on clinical and radiological findings. Only patients who underwent preoperative CT scanning within 24 h before emergency surgery were included in this study, because clinical evolution could disturb comparability between radiologic and surgical findings, when the interval is longer. Patient characteristics, preoperative findings, for example, Hinchey classification, Mannheim Peritonitis Index, specific findings on CT scan, and postoperative outcome were registered and analyzed.

A total of 158 consecutive patients underwent emergency surgery for perforated diverticulitis during the study period. Forty-six patients were excluded from analysis because they underwent emergency surgery without the performance of a preoperative CT. These patients were operated on based on clinical assessment only (*n* = 24), free intraperitoneal air on plain radiography (*n* = 16), or specific findings during ultrasound (*n* = 6). Another 37 patients were excluded because time of scanning was more than 24 h before surgery (median 3 days, range 2–50 days). The remaining 75 patients were included in the study, and the characteristics of these patients are listed in Table 2.

**Table 2:** Patient characteristics

Characteristics	
Gender (male/female)	30/45 (40/60 %)
Hospital (Erasmus/Maasstad)	38/37 (51/49 %)
Age	Median 63 years (range 23–89)
ASA	
I	13 (17 %)
II	25 (33 %)
III	27 (36 %)
IV	10 (13 %)
MPI	Median 19 (range 5–39)

Values in parentheses are percentages unless indicated otherwise

*ASA* American Society of Anaesthesiologist classification, *MPI* Mannheim Peritonitis Index

All preoperative CT scans were independently reviewed by a consultant radiologist and a senior radiology resident. Both were asked to classify disease severity according to the Hinchey classification (Table 1). Features recorded by the radiologist were, among others, thickness of bowel wall, number of diverticula, pericolic inflammation, stenosis, amount and location of free intraperitoneal air, fluid, and/or abscesses. Based on these features, they were asked to grade the severity of disease subjectively according to Hinchey’s classification. Both radiologists were blinded to the patients’ surgical and pathological findings at the time of CT review. If there was any discrepancy in the radiologists’ evaluations, a consultation between them took place so that they could come to a final agreement. Different types of CT scanners were used ranging from single-slice to 64-slice dual-source scanners. CT-examinations performed after 2001 at the Erasmus University and after 2006 at the Maasstad Hospital could be digitally analyzed. Different imaging protocols were used, and slice thickness varied between 3 and 8 mm. The contrast agent used was intravenous, oral, and/or rectal.

## Results

Sixty-six patients (88 %) received intravenous contrast, and 15 of them (20 %) received rectal contrast at the same time. Nine patients (12 %) underwent CT scanning without contrast. The location of the diverticular diseases was located in the sigmoid colon in 72 patients (96 %), in the descending colon in 16 patients (21 %), and in the transverse colon in 2 patients (3 %). Extra luminal air was found in 47 patients (64 %), and abscesses were found in 41 (55 %) patients. CT scanning showed bowel obstruction in one patient. No fistula formation was observed. Median colonic thickness was 9 mm (range 2–20 mm).

Comparison of findings during surgery (gold standard) and CT findings regarding Hinchey classification is shown in Table 4. The inter-observer agreement for scoring Hinchey was high with a discrepancy rate of 7 % (5/75). Final agreement was reached in the 5 cases that initially were differently scored by the radiologists. In all cases, the initial conclusion of the consultant radiologist was chosen.

Forty-eight of the 75 patients (64 %) were correctly staged by CT scanning in accordance with the Hinchey classification. Based on the results, sensitivity, specificity, positive predictive values (=precision of CT), and accuracy of CT were calculated for all stages of disease (Table 3). The use of rectal contrast did not significantly increase the accuracy of CT scanning (correctly staged with rectal contrast: 62 %, without rectal contrast: 73 %; *P* = 0.55). Stratifying the patients according to time intervals (within 12 h and between 12 and 24 h before surgery) did not change the result (correctly staged with 12 h: 62 %, between 12 and 24 h: 66 %: *P* = 0.81). In Table 4, distribution of specific CT features is listed for the different Hinchey stages found during surgery. Signs of diffuse intraperitoneal fluid on CT scans are not seen in Hinchey 1 and 2 patients (both 0 %). Nevertheless, free intraperitoneal fluid is not pathogmonomic for Hinchey 3 or 4 perforated diverticulitis, as it is only seen on CT scans in 38 and 56 % of cases, respectively. Intraperitoneal air in different amounts is found in almost all stages of perforated diverticulitis (75–100 %). The combination of diffuse free air and intra-abdominal fluid is strongly associated with Hinchey 3 and 4 (positive predictive value: 80 percent). The positive predictive value of CT scanning for perforated diverticulitis that requires surgical treatment (e.g., Hinchey 3 and 4) is 94 %. Unfortunately, the negative predictive value is only 61 %.

**Table 3:** Hinchey classification according to CT imaging compared to the true findings during surgery for perforated diverticulitis

	Hinchey classification at surgery			
	1	2	3	4
Hinchey classification according to CT scan				
1	13	*1* ^b^	*7* ^b^	–
2	*2* ^a^	9	*9* ^b^	–
3	*2* ^a^	–	16	–
4	–	–	*6* ^a^	10
Performance of CT scan				
Sensitivity (%)	76	90	42	100
Specificity (%)	86	83	95	91
Positive predictive value (%)	62	45	89	63
Negative predictive value (%)	93	98	61	100
Accuracy (%)	84	85	71	92

^a^overstaged

^b^understaged

The numbers that are underlined refer to the patients that are correctly classified by preoperative CT

The numbers that are italicized refer to the patients that are incorrectly classified by preoperative CT

*CT* computed tomography

**Table 4:** Specific computed tomography findings compared to true findings during surgery (Hinchey classification) in patients with perforated diverticulitis

Hinchey classification at surgery	Free intraperitoneal air (%)	Loculated gas bubbles (%)	Diffuse intraperitoneal fluid (%)	Abscess (%)	Pericolic fluid collection (%)
1	25	50	0	30	15
2	35	65	0	100	50
3	66	33	38	47	56
4	100	0	53	30	29

## Discussion

The optimal treatment strategy for perforated diverticulitis depends on the severity of disease classified according to Hinchey’s classification [18]. Ideally, perforated diverticulitis is adequately staged before surgery in order that the optimal treatment strategy (antibiotics, abscess drainage, surgery) can be chosen. In recent years, CT scanning has become the imaging modality of choice to determine the extent of the disease and surgeons tend to rely more frequently on the CT findings to decide upon further treatment.

**Fig. 1:**
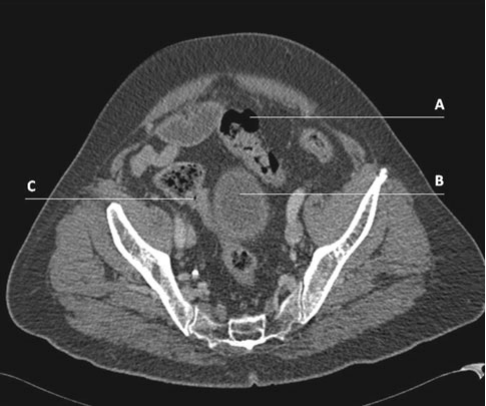
Preoperative CT image without evident signs of free fluid or generalized peritonitis of a patient who appeared to have Hinchey 3 perforated diverticulitis during surgery. *A* free air; *B* bladder; *C* colonic diverticulum

The present study shows that CT scanning has a high specificity for Hinchey 3 and 4 perforated diverticulitis (95 and 91 %, respectively). This means that when the radiologist diagnoses Hinchey 3 or 4 diverticulitis, this compares well with the true findings, and hence, emergency surgery is indicated. The positive predictive value for surgery is 94 %, which is excellent. Nevertheless, sensitivity for Hinchey 3 is low (42 %), meaning that a significant number of patients with Hinchey 3 diverticulitis are understaged (as Hinchey 1 or 2) by retrospective assessment of the CT scan. The main reason for this discrepancy was the relatively small amount of free intra-abdominal pus found during surgery. This can easily be missed on an emergency CT scan (Fig. 1). Another reason for the relatively high number of misclassifications of Hinchey 3 perforated diverticulitis by preoperative CT scanning could be rupture of a diverticular abscess, in which Hinchey 2 perforated diverticulitis found on the CT scan has proceeded toward Hinchey 3 at the time of surgery [19]. It is therefore possible that future patients who undergo CT scanning are classified as Hinchey 1 or 2 perforated diverticulitis and are treated according to these CT findings (that is conservatively), are in reality Hinchey 3 patients (*n* = 16/41; 39 % of Hinchey 1 and 2 cases; Table 3), and should have been treated surgically. It seems that only Hinchey 4 perforated diverticulitis is excellently staged by CT scanning. The conclusion after the radiologists’ report will always be that emergency surgery is indicated in these patients. Due to the low sensitivity of CT scanning in Hinchey 3 patients, the predictive value of CT for conservative treatment is only 61 %.

The inter-observer agreement for scoring Hinchey was high. In 5 cases, the consultant radiologist convinced the resident to revise her conclusion. In daily practice, and especially during night shifts, the CT scan is first read by the radiology resident. If necessary, the original reading is changed by the consultant radiologist, who will see the CT only the day after. The relative inexperience by the residents could lead to over- or undertreating a patient with perforated diverticulitis who undergoes a CT scan. Although in this study overtreatment or undertreatment was not caused by this phenomenon, we recommend a dedicated consultant radiologist to read all CT scans performed on patients in this category.

Lohrmann et al. [14] previously investigated the value of CT scanning in diverticular disease. They stated that CT scanning correctly determined Hinchey stage in 93 % of patients. Unfortunately, only 7 patients were found to have Hinchey 3 or 4 perforated diverticulitis (CT sensitivity of 71 % in this subgroup). This suggests that the study was based on a heterogeneous group of patients, only a few of whom had perforated diverticulitis with peritonitis.

Ritz et al. [15] conclude in their study on 204 patients who had undergone surgery for diverticular disease that CT scanning is an accurate modality for staging this disease. The positive predictive value of CT scanning compares well with the results of this present study, especially the positive predictive value of perforated diverticulitis Hinchey 3 and 4 (100 and 94 %). Unfortunately, surgery was performed within 24 h after CT scanning in only 42 patients (21 %). In all other patients, elective surgery was performed after a mean of almost 7 days of initial conservative therapy with antibiotics or percutaneous abscess drainage. No new CT scan was performed prior to elective surgery; hence, clinical evolution could have disturbed comparability between radiologic and surgical findings.

The present study exclusively covers patients with perforated diverticulitis. Nevertheless, 36 % of the patients studied who underwent surgery appeared to have Hinchey 1 or 2 during surgery (Table 4; *n* = 27). Twenty-five of these patients were ‘proven’ Hinchey 1 or 2 by preoperative CT scanning, but the indication for emergency surgical treatment was set by the surgeon on call who probably doubted the CT report in combination with the clinical symptoms (sepsis, acute abdomen). These patients could conceivably be treated conservatively instead if preoperative (CT) assessment had 100 % accuracy. Even if subjective ‘clinical’ signs of acute abdomen are present or objective findings of small amounts of free air are present on CT (75 % of Hinchey 1 patients and 90 % of Hinchey 2 patients; Table 3), true Hinchey 1 and 2 patients can be treated conservatively with antibiotics and analgesics [18]. If this conservative treatment fails, surgical intervention is indicated.

The combination of free air and intra-abdominal fluid seen on the CT scan correlated well with Hinchey 3 and 4 perforated diverticulitis as found during surgery, and these are the main findings the radiologists used to for the CT-based diagnosis of Hinchey 3 or 4. Only very few patients with a CT scan diagnosis of Hinchey 3 or 4 diverticulitis appear to have a stage of disease during surgery that might have been treated successfully without surgery. In other words, large amounts of free air and free fluid are indications for emergency surgery.

Preoperative differentiation between Hinchey stage 3 and 4 is not very important, as both need emergency surgical treatment. Nevertheless, it could be useful in deciding on the surgical approach [5]. In case of purulent peritonitis (Hinchey 3), laparoscopic peritoneal lavage and drainage without resection of the affected bowel segment has shown excellent results [10]. In case of fecal peritonitis, laparotomy is recommended for resection of the affected bowel segment [5]. Unfortunately, the present study shows that preoperative differentiation between Hinchey 3 and Hinchey 4 is not possible with CT scanning. It is therefore advisable to perform diagnostic laparoscopy, when the CT scan shows large amounts of free air and fluid (CT Hinchey 3/4). When purulent peritonitis is found, laparoscopic treatment could be performed. In case of fecal spill, conversion toward laparotomy is indicated.

CT technology has evolved rapidly in the past decades and will continue to do so in the future. In previous studies, CT scanning could only visualize bowel wall discontinuity in a minority of patients with proven bowel perforation [14]. Thanks to advances in technology, multidetector row CT scanners are able to visualize the site and size of the perforation more accurately [20–22]. This additional information would be helpful in deciding on the appropriate surgical technique. In Hinchey 3 perforated diverticulitis, most of times the perforation has been sealed by omentum. In case of Hinchey 4 diverticulitis, an overt perforation is found, causing a fecal spillage.

## Conclusions

Current CT scanning does not seem to suffice to accurately predict the severity of perforated diverticulitis according to Hinchey’s classification [21]. Nevertheless, specific findings on CT like the combination of a large amount of free intraperitoneal air and diffuse intraperitoneal fluid are a good predictor for Hinchey 3 or 4 diverticulitis and mandate surgical intervention. Diagnostic laparoscopy is advised in these patients to distinguish between purulent or fecal peritonitis. To date, distinction between Hinchey 3 and 4 with preoperative CT scanning is not possible. Diagnosis of Hinchey 1 or 2 perforated diverticulitis after CT assessment is not reliable, as 39 % of these patients are in fact Hinchey 3 patients for whom surgery is indicated. In the absence of free intraperitoneal air, conservative treatment is justifiable. A prospective study is warranted to confirm our statements.
